# Mental Health Status, Risk and Protective Factors for Healthcare Staff Prior to the First Major COVID-19 Outbreak in Western Australia

**DOI:** 10.3389/ijph.2023.1606102

**Published:** 2023-09-05

**Authors:** Elizabeth A. Newnham, Enrique L. P. Mergelsberg, Jessica Tearne, Peter McEvoy, Susanne Stanley, Antonio Celenza, Hyranthi Kavanagh, Teresa Stevenson, Nahal Mavaddat, Gavin Demore, Sean Hood

**Affiliations:** ^1^ School of Population Health, Curtin University, Perth, WA, Australia; ^2^ Curtin enAble Institute, Perth, WA, Australia; ^3^ François-Xavier Bagnoud Center for Health and Human Rights, Harvard T.H. Chan School of Public Health, Boston, MA, United States; ^4^ EnBerg Analytics, Perth, WA, Australia; ^5^ Department of Clinical Psychology and Clinical Neuropsychology, Fiona Stanley Hospital, Perth, WA, Australia; ^6^ State Major Trauma Unit, Royal Perth Hospital, Perth, WA, Australia; ^7^ Centre for Clinical Interventions, North Metropolitan Health Service, Perth, WA, Australia; ^8^ Division of Psychiatry, School of Medicine, The University of Western Australia, Perth, WA, Australia; ^9^ Emergency Medicine, School of Medicine, University of Western Australia, Perth, WA, Australia; ^10^ Emergency Department, Sir Charles Gairdner Hospital, Perth, WA, Australia; ^11^ Peel and Rockingham Kwinana Mental Health Service, Rockingham, WA, Australia; ^12^ Discipline of General Practice, School of Medicine, University of Western Australia, Perth, WA, Australia; ^13^ Western Australia Country Health Service, Perth, WA, Australia; ^14^ Sir Charles Gairdner Hospital Mental Health Unit, North Metropolitan Health Service Mental Health, Public Health and Dental Services (MHPHDS), Perth, WA, Australia

**Keywords:** COVID-19, healthcare workers, PTSD, depression, anxiety, Health EDRM, Australia

## Abstract

**Objectives:** Western Australia’s unique public health response delayed the first wave of community COVID-19 transmission for 2 years. We aimed to determine the status of post-traumatic stress (PTSS), depressive, and anxiety symptoms among healthcare staff in major tertiary hospitals, together with associated risk and protective factors prior to the first substantial outbreak of COVID-19.

**Methods:** A cross-sectional study was conducted with 431 healthcare staff immediately prior to the Western Australian border re-opening in 2022. Staff were recruited via notices in email newsletters, at four tertiary hospitals and a public mental health clinic in metropolitan Perth. Validated and original questionnaires were administered via Qualtrics.

**Results:** Moderate levels of PTSS (22.3%), depression (21.9%), and anxiety (25.9%) were reported. Pathway analyses indicated that sleep difficulties, workplace stressors, and infectious disease training were associated with higher PTSS, depression and anxiety symptoms, and younger age was associated with higher levels of depression and anxiety. Nursing roles were associated with higher PTSS. Social support and workplace support were associated with lower levels of depression and anxiety but were not associated with PTSS.

**Conclusion:** The findings illustrate high levels of resilience, but indicate a need for structural supports within the health system to foster staff mental health prior to the onset of emergencies.

## Introduction

Western Australia’s trajectory during the COVID-19 pandemic provides a novel opportunity to investigate mental health status among healthcare workers in anticipation of a large-scale health emergency. Low population density, geographic isolation and a strategic state-wide public health response in Western Australia placed the state in a rare position to minimise the transmission of SARS-COV-2 (COVID-19) for the first 2 years of the global pandemic. By limiting international and interstate arrivals, implementing strict quarantine procedures, establishing rapid city-wide lockdowns, and encouraging physical distancing, the government enabled sufficient time for more than 95% of the population (over 16 years) to have received two vaccines and 66% to have had three vaccine doses by 3rd March 2022, when the international and state borders were officially reopened [1, 2]. At the time, the Western Australian healthcare system had not experienced the immense burden of COVID-19 cases evident internationally. Prior to the border re-opening in 2022, 70 people had been hospitalised and 11 people had died from COVID-19 in Western Australia in total [1, 2]. Accordingly, the WA health system had additional time to prepare for the pandemic response with knowledge gained from global health settings.

The COVID-19 pandemic has had significant impacts on healthcare professionals’ mental health internationally. Recent cross-sectional studies indicate significantly elevated rates of post-traumatic stress symptoms, depression, and anxiety among healthcare staff responding to the COVID-19 pandemic [3, 4]. Poorer mental health status has been associated with a variety of individual, professional and workplace factors. First, nurses’ professional positions compared to doctors and other health professionals have been associated with poorer mental health status, likely due to the frontline roles, changing demands, and exposure risks that nurses have experienced during the pandemic [5–7]. Second, physical health risks, such as greater exposure to COVID-19 patients in the healthcare setting [8], safety protection concerns at work [8, 9], worry about infecting family members or blame from colleagues for not taking adequate precautions [10, 11], and higher levels of health-related fears [8] place healthcare workers at higher risk of mental health concerns. Third, poor sleep quality and duration has also been associated with poor mental health outcomes [12], but the need for further research on the role of sleep during COVID-19 has been highlighted [13]. Consistent with an empirical gap in disaster and pandemic research [14], fewer studies have investigated protective factors associated with mental health outcomes among healthcare staff. An emerging evidence base suggests that adaptive coping strategies [15, 16], social support [17, 18], workplace supports [19], and additional infectious disease training [16, 17] may play important roles in reducing mental health concerns for healthcare staff.

Western Australian healthcare workers witnessed the catastrophic outcomes of the global pandemic from afar, whilst preparing for the arrival of a first wave in 2022. Despite the delay in large-scale outbreaks of COVID-19 cases in the community, healthcare staff were under significant strain with shortages in staff and rapid policy changes to prepare for the onset of the pandemic [20, 21]. Changes to personal protective equipment policies, training requirements, staff schedules and roles were rapidly implemented. Following 2 years of international and state border closures with limited flights and hotel quarantine requirements (although short periods of interstate travel were allowed when case numbers where low), March 2022 presented the first opportunity for travel and migration.

The unique context in Western Australia prior to March 2022 provided an opportunity to investigate the mental health impacts for healthcare workers preparing for an impending pandemic. Understanding the factors associated with healthcare workers’ mental health in anticipation of a large-scale health emergency will inform avenues for health system preparedness and maximise sustainability of the workforce. Accordingly, the present study captured healthcare workers’ mental health data in the 3 months leading up to the Western Australian 2022 interstate and international border re-opening. We aimed to determine the status of post-traumatic stress, depressive, and anxiety symptoms among healthcare staff in major tertiary hospitals and clinics in Perth, Western Australia, and the associated individual, social and workplace risk and protective factors prior to a substantial COVID-19 outbreak. Building on prior findings among studies of healthcare professionals in settings affected by COVID-19, it was hypothesized that:

First (H_1_): Mental health status would differ by profession type, with nurses reporting higher levels of mental health concerns, and lower levels reported by doctors, allied health, executives and management.

Second (H_2_): Workplace stressors would be positively associated with PTSS, depression, and anxiety symptoms for all profession groups.

Third (H_3_): Sleep difficulties would be positively associated with PTSS, depression and anxiety symptoms for all profession groups.

Fourth (H_4_): Resilient coping, social support, infectious disease training, and workplace supports would be negatively associated with PTSS, depression and anxiety symptoms among all healthcare workers.

## Methods

### Participants and Procedure

This study presents the cross-sectional baseline survey data for a larger longitudinal study of secondary and tertiary hospital staff wellbeing and mental health during the COVID-19 pandemic. All healthcare staff (including doctors, nurses, midwives, allied health professionals, auxiliary staff, executives and management) employed at four major tertiary hospitals and a large public mental health service in metropolitan Perth, WA, were eligible to participate. Hospitals and clinics in Perth are managed by overarching Health Service Providers (HSPs) through the Department of Health. The study was promoted via a small advertisement in the all-staff email newsletter at some HSPs, online flyers distributed through HSP communications, at hospital-wide forums and at team meetings attended by investigators on the project. In one HSP, two follow-up reminders were included in all-staff newsletters. Hospital executive and management staff were not involved in the recruitment or knowledgeable about participation of staff. Participants were recruited between 30 November 2021 and 7 March 2022, and the questionnaire was accessed online. All respondents provided informed consent to participate. The data were deidentified. Participants created a private unique code to link data at later stages. Respondents were asked to provide their email address to enable follow-up, which was stored separately to the data. Ethics approvals were granted by the Department of Health North Metropolitan Area Mental Health Services Human Research Ethics Committee (RGS0000004034).

### Measures

The survey was hosted online via Qualtrics [22] and comprised validated measures of psychological symptoms, validated and original measures of risk and protective factors, and a checklist of mental health supports.

#### Demographics

Demographic items included age, gender, profession, department, work setting and employment status. We measured respondents’ prior experience working with infectious disease (one item; yes = 1, no = 0), direct and indirect COVID-19 exposure in their current workplace (sum of three items; yes = 1, no = 0), and training (one item; yes = 1, no = 0, with a follow-up question when answered ‘yes’ to assess when they had the training: pre-, during or both pre- and during COVID).

#### Workplace Factors

Work related stressors and supports scales were developed by the study team, based on a review of the relevant literature, consultations with healthcare staff (*n* = 7), and team expertise. The scales were designed to capture specific stressors and supports highlighted as relevant to healthcare staff in the early stages of the pandemic (please see Supplementary Material for scale). Workplace stressors (12 items including concerns about exposure to COVID-19 at work, financial security, changes to workload, risk of infecting people in your household with COVID-19, and risk of contracting other infectious illnesses) were measured on a 5-point Likert scale ranging from “Not at all concerned” [1] to “Very concerned” [5]. Sum scores were calculated, higher sum scores indicated higher levels of work-related stress (range: 12–60). Internal consistency was high (*α* = .88). McDonald’s Omega coefficient (ωt) for the workplace stressors scale was 0.900 (above 0.700 is sufficient reliability).

Satisfaction with work-related support (11 items, including the timely provision of information, managerial support, clarity of instructions, and access to personal protective equipment), was measured via a 5-point Likert scale assessing level of satisfaction respectively, from “Not at all satisfied” [1] to “Very satisfied” [5] (please see Supplementary Material for scale). Internal consistency was high (*α* = .91, ωt = 0.921). Sum scores were calculated (range: 11–55), with high scores indicating high level of satisfaction with the provision of support.

#### Protective Factors

Coping strategies were assessed with the 4-item Brief Resilient Coping Scale questionnaire (BRCS) [23], rated on a 5-point Likert scale ranging from “Does not describe me” [1] to “Describes me very well” [5] with good internal consistency (*α* = .71). We calculated a sum score (ranging 4–20) with higher scores indicating better coping. Social support was measured with the 3-item Oslo Social Support Scale [24] (*α* = .77). A sum score was calculated (range: 3–14) with higher scores indicating stronger social support. The average inter-item correlation was good (*Mr* = .55) with *a* = .77.

#### Sleep Quality

Sleep difficulties were assessed with the Sleep Condition Indicator (SCI; 25). A sum score was calculated ranging from 0–32, higher scores indicating more sleep problems. We applied a cut-off at a score of 16 to report prevalence of probable insomnia disorder, as this has shown to have a sensitivity of 89% and 82% specificity, respectively [25]. The average inter-item correlation was good (*Mr* = .52) with *a* = .88.

#### Post-Traumatic Stress Symptoms

The PCL-5 Trauma Checklist was administered to measure trauma exposure, and post-traumatic stress symptoms were assessed using the Primary Care PTSD Screen for DSM-5 (PC-PTSD-5; 26). A sum score was calculated for those who experienced trauma on 5-items asking to rate whether they experienced symptoms (e.g., having nightmares: yes = 1, no = 0) related to the experience they listed as most distressing (range: 0–5, higher scores indicated higher levels of PTSS). The average inter-item correlation was ideal (*Mr* = .31) with *a* = .69. Prevalence of probable clinical levels of PTSS was reported using a cut-off point of 4 points as recommended [26, 27]. For analyses, trauma exposure was coded as trauma [0 = none, 1 = infectious disease, 2 = work-related trauma (physical/sexual assault at work, death of patient and medical litigation), 3 = not work-related trauma (all other exposures)].

#### Depressive Symptoms

Depression was assessed with the Patient Health Questionnaire (PHQ-9; [28]), scoring each of the nine DSM-5 depression criteria on a 4-point Likert scale ranging from “Not at all” (0) to “Nearly every day” [3]. The average inter-item correlation was ideal (*Mr* = .46) with *a* = .88. Sum scores were calculated (range: 0–27), with higher scores indicating higher levels of depression. Scores of 5, 10, 15, and 20 were taken as the cut-off points for mild, moderate, moderately severe, and severe depression respectively, consistent with prior research in a comparative sample [10].

#### Anxiety Symptoms

Anxiety was assessed using the Generalised Anxiety Disorder Questionnaire (GAD-7; [29, 30]) using 4 response options ranging from “Not at all” (0) to “Nearly every day” [3]. The average inter-item correlation was good (*Mr* = .61) with *a* = .92. Sum scores were calculated (range: 0–21) and scores of 5, 10, and 15 were taken as the cut-off points for mild, moderate, and severe anxiety, respectively. consistent with prior research [10].

#### Access to Psychological Services

Access to psychological and wellbeing services were assessed with an 18-item checklist that included general services (e.g., accessing a General Practitioner, psychologist, online information, listening to a podcast) as well as services delivered in specific hospital settings (such as a drop-in wellbeing hub) nominated by clinicians in the research team. For each item, participants were asked to tick whether they had accessed the service, and for those they had, rate their satisfaction on a Likert scale of 5 (Highly Satisfied) to 1 (Highly Dissatisfied).

### Data Analysis

Data were exported from Qualtrics and uploaded in R statistics [31]. We reported descriptives, before completing a missing values analysis using Little’s MCAR test [32] with the *naniar* package. In cases of random missingness we imputed the data for participants who had less than 50% missingness using the regression imputation method with the *impute_lm* function from the *simputation* package in R [33] as recommended by Newman [34]. We ran Pearson correlations between all measured variables to assess associations and assured the lack of multicollinearity, and conducted a pathway analysis to test whether demographic variables, risk factors and protective factors were associated with PTSS, depression, and anxiety symptoms.

## Results

### Sample Characteristics

A total of 563 individuals accessed the online survey, of these 533 provided consent to participate. Completed data were available for *n* = 431 (102 participants closed Qualtrics before completing measures). Mean age was 42.4 (SD = 11.9, range = 21–71). Most participants (81%) identified as women, 18% as men, 0.5% as another gender, and 0.5% preferred not to say. Occupational characteristics of the sample are shown in Table 1. Prior experience with infectious disease outbreaks was reported by 21% (*n* = 91) of the sample, and only 39% (*n* = 167) reported having received training in epidemic/pandemic infectious disease management. Of those, most had received training only during the COVID-19 pandemic (*n* = 84, 51%), or both before and during the pandemic (*n* = 66, 40%). Sixteen participants (9.6%) reported only having received training prior to the COVID-19 pandemic.

**TABLE 1:** Demographic characteristics of all participating healthcare workers (N = 431) and by probable mental health status. Western Australia, 2021–2022.

Variable	All	Probable PTSD, N = 78^a^	Probable depression, N = 94^a^	Probable anxiety, N = 102^a^
	N = 431^1^			
Age (M, SD)	42.4 (11.9)	42.1 (11.7)	40.1 (12.1)	39.3 (11.7)
(Missing)	31	6	3	4
Age categories				
<30 years	82 (20%)	12 (17%)	25 (27%)	27 (28%)
>70 years	1 (0.2%)		0 (0%)	0 (0%)
31–40 years	101 (25%)	22 (31%)	25 (27%)	28 (29%)
41–50 years	100 (25%)	17 (24%)	18 (20%)	21 (21%)
51–60 years	91 (23%)	17 (24%)	19 (21%)	18 (18%)
61–70 years	25 (6.2%)	4 (5.6%)	4 (4.4%)	4 (4.1%)
(Missing)	31	6	3	4
Gender				
Female	351 (81%)	65 (83%)	80 (85%)	89 (87%)
Male	76 (18%)	13 (17%)	13 (14%)	12 (12%)
Other	2 (0.5%)	0 (0%)	1 (1.1%)	1 (1.0%)
Prefer not to say	2 (0.5%)	0 (0%)	0 (0%)	0 (0%)
Employment				
Full-Time	292 (68%)	56 (72%)	73 (78%)	78 (77%)
Part-Time	124 (29%)	19 (24%)	19 (20%)	20 (20%)
Casual	14 (3.3%)	3 (3.8%)	2 (2.1%)	3 (3.0%)
(Missing)	1	0	0	1
Profession				
Administrative	46 (11%)	9 (12%)	15 (16%)	11 (11%)
Allied Health	121 (28%)	16 (21%)	20 (21%)	19 (19%)
Doctor	37 (8.6%)	1 (1.3%)	3 (3.2%)	4 (3.9%)
Executive	7 (1.6%)	1 (1.3%)	0 (0%)	2 (2.0%)
Managerial	35 (8.1%)	7 (9.0%)	9 (9.6%)	9 (8.8%)
Midwife	9 (2.1%)	1 (1.3%)	3 (3.2%)	2 (2.0%)
Nurse	175 (41%)	43 (55%)	44 (47%)	55 (54%)
(Missing)	1	0	0	0
Area				
Multiple Areas	85 (22%)	19 (27%)	21 (25%)	26 (28%)
Singular Area	305 (78%)	52 (73%)	62 (75%)	68 (72%)
(Missing)	41	7	11	8
Discipline				
Administration	69 (16%)	13 (17%)	20 (21%)	16 (16%)
Anaesthetics	15 (3.5%)	1 (1.3%)	3 (3.2%)	0 (0%)
Dietetics	7 (1.6%)	1 (1.3%)	2 (2.1%)	2 (2.0%)
Emergency	46 (11%)	12 (15%)	11 (12%)	15 (15%)
General Practice	5 (1.2%)	0 (0%)	0 (0%)	0 (0%)
ICU	8 (1.9%)	4 (5.1%)	2 (2.1%)	4 (3.9%)
Internal Medicine	14 (3.2%)	2 (2.6%)	4 (4.3%)	7 (6.9%)
Midwifery	10 (2.3%)	1 (1.3%)	2 (2.1%)	2 (2.0%)
Nursing	126 (29%)	28 (36%)	33 (35%)	37 (36%)
Obstetrician and Gynaecology	3 (0.7%)	0 (0%)	0 (0%)	0 (0%)
Occupational Therapy and Physiotherapy	27 (6.3%)	3 (3.8%)	6 (6.4%)	5 (4.9%)
Paediatrics	12 (2.8%)	4 (5.1%)	4 (4.3%)	5 (4.9%)
Psychiatry	65 (15%)	9 (12%)	6 (6.4%)	10 (9.8%)
Psychology	32 (7.4%)	3 (3.8%)	3 (3.2%)	2 (2.0%)
Pharmacy	11 (2.6%)	1 (1.3%)	1 (1.1%)	2 (2.0%)
Rehabilitation	18 (4.2%)	4 (5.1%)	4 (4.3%)	2 (2.0%)
Respiratory	10 (2.3%)	3 (3.8%)	3 (3.2%)	4 (3.9%)
Speech Pathology	7 (1.6%)	1 (1.3%)	1 (1.1%)	2 (2.0%)
Surgical	27 (6.3%)	6 (7.7%)	8 (8.5%)	12 (12%)
Laboratory or Imaging	9 (2.1%)	2 (2.6%)	2 (2.1%)	1 (1.0%)
Oncology	3 (0.7%)	0 (0%)	0 (0%)	0 (0%)
Social Support Work	29 (6.7%)	5 (6.4%)	6 (6.4%)	4 (3.9%)
Research or Education	19 (4.4%)	2 (2.6%)	7 (7.4%)	7 (6.9%)
Other Area	12 (2.8%)	3 (3.8%)	5 (5.3%)	5 (4.9%)
Setting				
Multiple Settings	24 (6.3%)	5 (7.2%)	6 (7.8%)	3 (3.3%)
Singular Setting	354 (94%)	64 (93%)	71 (92%)	87 (97%)
(Missing)	53	9	17	12
Setting details^b^				
ED	57 (13%)	15 (19%)	11 (12%)	14 (14%)
Inpatients	130 (30%)	28 (36%)	34 (36%)	40 (39%)
Outpatients	87 (20%)	7 (9.0%)	11 (12%)	15 (15%)
Both in- and outpatients	114 (26%)	20 (26%)	24 (26%)	21 (21%)
Community	15 (3.5%)	4 (5.1%)	3 (3.2%)	3 (2.9%)
Office	46 (11%)	9 (12%)	12 (13%)	8 (7.8%)
Other Setting	16 (3.7%)	3 (3.8%)	7 (7.4%)	4 (3.9%)
Training epidemic/pandemic infectious disease outbreaks				
Not received training	261 (61%)	34 (44%)	48 (51%)	53 (52%)
Received training	167 (39%)	44 (56%)	46 (49%)	49 (48%)
(Missing)	3		0	
Time of training				
Both before and during COVID	66 (40%)	19 (43%)	15 (33%)	16 (33%)
During COVID	84 (51%)	20 (45%)	26 (57%)	27 (55%)
Pre-COVID	16 (9.6%)	5 (11%)	5 (11%)	6 (12%)
(Missing)	265	34	48	53
Exposure				
Exposure to COVID-19	212 (50%)	44 (56%)	50 (53%)	56 (55%)
No exposure to COVID-19	215 (50%)	34 (44%)	44 (47%)	46 (45%)
(Missing)	4			

^a^ Mean (SD) for continuous variables and n (%) for categorical variables.

^b^ Does not add up to 100% as participants were allowed to click multiple options. Other disciplines reported included allied health, specialty services, nuclear medicine, palliative care, geriatric care. Other settings included classrooms (education), kitchen, pharmacy, and population health.

A large majority of the sample reported prior lifetime trauma exposure (*n* = 333, 85%), with an average of 3.3 types of trauma exposures per person (SD = 2.1). The most frequently reported trauma exposures were the unexpected death of a patient (*n* = 199, 37%), physical assault in the workplace (*n* = 174, 33%), experience of a life threatening illness (*n* = 156, 29%), infectious disease outbreak (*n* = 124, 23%), physical assault external to work (*n* = 76, 14%), natural disaster (*n* = 71, 13%), serious accident (*n* = 66, 12%), medical litigation (*n* = 65, 12%), sexual assault external to work (*n* = 62, 12%), and child abuse (*n* = 62, 12%). Of the respondents who completed the Sleep Condition Indicator, half reported difficulties with sleep (*n* = 185, 50%).

### Missing Values Analysis

The missing values analysis showed that the data were missing completely at random (MCAR), *X* ^*2*^ (7818) = 4,770, *p* = 1.00. The average missing values among respondents was 18.9%, with an average of 81.1% complete. Twenty-six participants had more than 70% missing and 19 participants had between 50% and 69% missingness. The remaining (and included) participants had between 0% and 33% missingness with an average of 1.4% missingness. Hence missing data were imputed for those with less than 50% missingness (*n* = 27 deleted case-wise), leaving N = 404. Of these, *n* = 247 (61.1%) had a complete data set. In 17 cases, imputation failed due to missing data in predictor variables needed for imputation, and so they were omitted from analysis. The final dataset for the regression models comprised 387 cases.

### Mental Health Status

Mental health symptom reports for the total sample indicated: probable PTSS (*n* = 78, 22.3%), depression in the severe (*n* = 5, 1.2%), moderate severe (*n* = 24, 5.6%), moderate (*n* = 65, 15.1%), and mild range (*n* = 113, 26.2%), and anxiety in the severe (*n* = 33, 8.35%), moderate (*n* = 69, 17.5%) and mild range (*n* = 106, 26.8%). We examined differences in psychological symptoms by discipline, shown in Table 2. Pairwise comparisons adjusted by Bonferroni showed that nurses reported significantly higher PTSS, depression, and anxiety scores, compared to doctors (*p* = .006, *p* = .038, and *p* = .006, respectively). Nurses also reported significantly higher PTSS compared to Allied Health staff (*p* = .015). Executive and administrative staff reported marginally higher depression scores compared to doctors (*p* = .078). All other differences were not significant (*p* > .210).

**TABLE 2:** Mental health status of healthcare workers in WA per profession (N = 430) Western Australia, 2021–2022.

	Executive and administrative (N = 88)^a^	Doctors (N = 37)^a^	Nurses and midwives (N = 184)^a^	Allied health (N = 121)^a^	Difference between profession groups (*p-*value)^b^
Post-traumatic Stress Symptoms	1.87 (1.70)	1.38 (1.31)	2.48 (1.65)	1.91 (1.59)	.004
Probable PTSD	17 (37%)	1 (4.2%)	44 (30%)	16 (21%)	
Probable no PTSD	29 (63%)	23 (96%)	102 (70%)	59 (79%)	
(Missing)	42	13	38	47	
Depression	6.7 (5.6)	3.7 (4.1)	6.7 (5.2)	5.1 (4.6)	.001
Probable severe depression	2 (2.3%)	0 (0%)	2 (1.1%)	1 (0.8%)	
Probable moderate severe depression	4 (4.5%)	1 (2.7%)	14 (7.6%)	5 (4.1%)	
Probable moderate depression	18 (20%)	2 (5.4%)	31 (17%)	14 (12%)	
Probable mild depression	16 (18%)	7 (19%)	58 (32%)	31 (26%)	
Probable no depression	48 (55%)	27 (73%)	79 (43%)	70 (58%)	
(Missing)	13	2	14	7	
Anxiety	6.2 (5.5)	3.7 (4.0)	7.1 (5.5)	5.5 (4.8)	.002
Probable severe anxiety	8 (11%)	1 (2.9%)	18 (11%)	6 (5.3%)	
Probable moderate anxiety	14 (19%)	3 (8.6%)	39 (23%)	13 (11%)	
Probable mild anxiety	17 (23%)	6 (17%)	45 (26%)	37 (32%)	
Probable no anxiety	36 (48%)	25 (71%)	68 (40%)	58 (51%)	
(Missing)	13	2	14	7	

^a^ n (%); Mean (SD).

^b^ Fisher’s exact test.

### Risk and Protective Factors for Mental Health

Pearson correlations were conducted on the final sample (*n* = 387, see Supplementary Material). We found significant correlations with all adverse mental health outcomes and the measured predictor variables (i.e., age, gender, profession, exposure to COVID-19, work related stressors, trauma, sleep quality, work related support, coping strategies, and social support). The highest correlations were between insomnia and the adverse mental health outcomes (PTSS: *r* = .36, *p* < .001; depression *r* = .53, *p* < .001; anxiety: *r* = .51, *p* < .001). The smallest significant correlations were found with previous experience with infectious diseases variables, which only correlated with PTSS, age and profession (*r* = .11, *p* < .050, *r* = .11, *p* < .050 and *r* = .18, *p* < .001, respectively).

**FIGURE 1:**
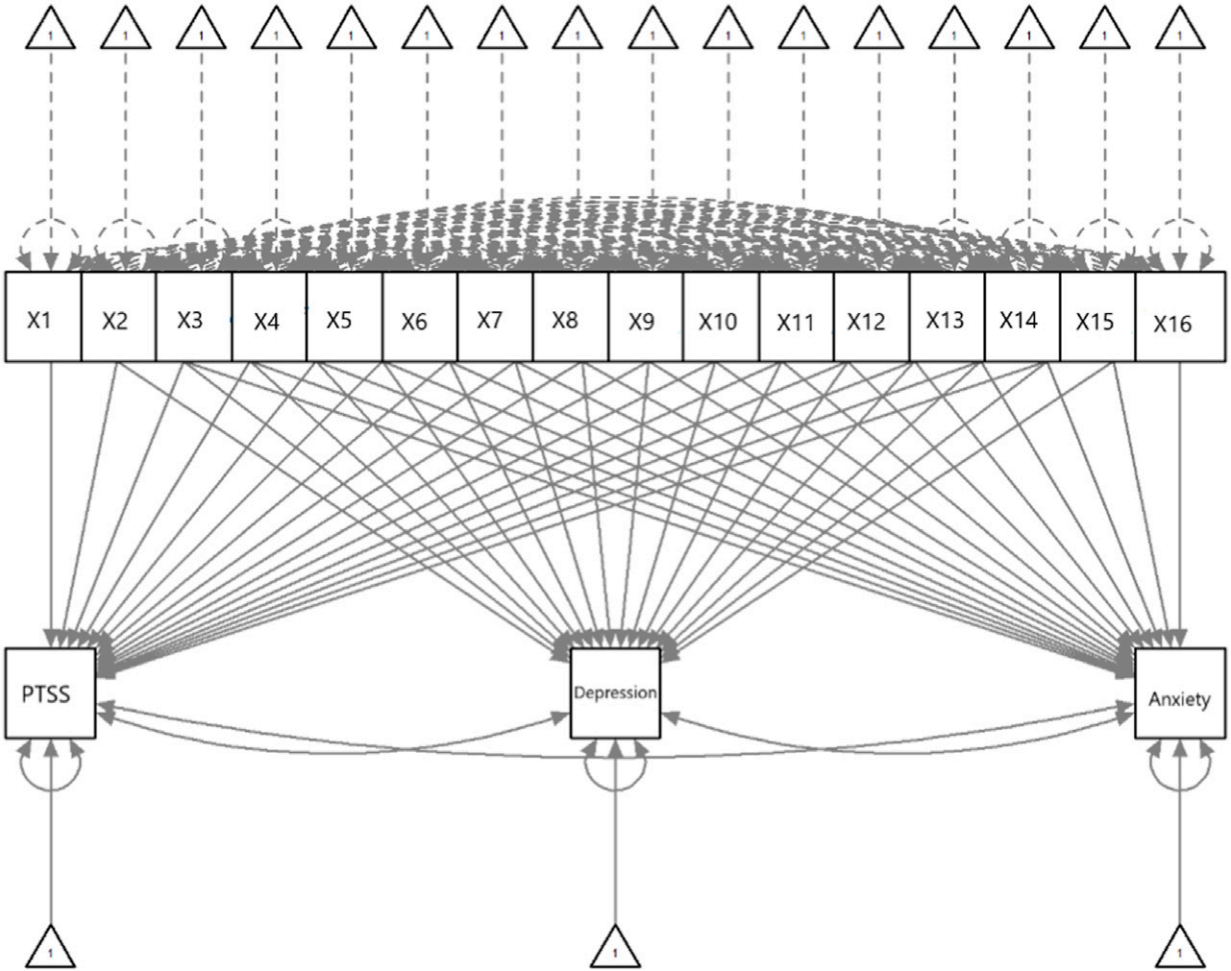
Diagram of pathway analysis assessing causal effects of risk and protector factors and mental health outcomes. Western Australia, 2021–2022.

Model fit of the pathway model (Figure 1), along with unstandardized coefficients with standard errors and 95% confidence intervals are shown in Table 3. The model had excellent fit and showed that younger healthcare workers reported higher levels of depression and anxiety, but that gender was not associated with adverse mental health outcomes. The largest effect was found for difficulties with sleep, which was associated with higher levels of PTSS, depressive and anxiety symptoms. Further, we found that a greater number of workplace stressors and having received infectious disease training were associated with higher levels of PTSS, depression and anxiety symptoms. Nurses were most likely to report PTSS than other disciplines, and prior experience with infectious disease outbreaks was associated with lower levels of depressive symptoms. Contrary to expectation, there was no association between exposure to patients with COVID-19 and adverse mental health outcomes for healthcare staff. Social support and workplace support were associated with lower levels of depression and anxiety but were not associated with lower levels of PTSS.

**TABLE 3:** Regression coefficients of associative paths of PTSS, Depression and Anxiety (N = 387) Western Australia, 2021–2022.

		PTSS		Depression		Anxiety	
		*β* (SE)	95%CI	*β* (SE)	95% CI	β (SE)	95% CI
	Intercept	−2.655 (1.435)	−5.468, 0.159	2.417 (4.003)	−5.429, 10.263	−1.815 (4.142)	−9.034, 6.304
_X1_	Age	−0.003 (0.007)	−0.016, 0.010	−0.065 (0.019)***	−0.101, −0.029	−0.086 (0.019)***	−0.123, −0.048
_X2_	Gender (female)	0.031 (0.205)	−0.371, 0.434	0.338 (0.573)	−0.785, 1.460	1.024 (0.593)	−0.138, 2.185
_X3_	Employment (full-time)	0.080 (0.158)	−0.230, 0.391	0.318 (0.442)	−0.549, 1.184	0.545 (0.457)	−0.351, 1.441
_X4_	Profession (managerial)	0.564 (0.318)	−0.060, 1.188	1.715 (0.888) ∼	−0.025, 3.455	1.492 (0.919)	−0.308, 3.293
_X5_	Profession (allied health)	0.408 (0.286)	−0.153, 0.969	0.772 (0.798)	−0.793, 2.336	1.050 (0.826)	−0.569, 2.669
_X6_	Profession (nurse/midwife)	0.616 (0.281)*	0.066, 1.167	0.608 (0.783)	−0.927, 2.143	1.096 (0.810)	−0.493, 2.684
_X7_	Setting (forefront)	0.041 (0.210)	−0.370, 0.452	−0.221 (0.585)	−1.367, 0.925	0.775 (0.605)	−0.411, 1.961
_X8_	Insomnia	0.040 (0.010)***	0.020, 0.061	0.249 (0.029)***	0.192, 0.305	0.241 (0.030)***	0.183, 0.299
_X9_	Trauma	0.069 (0.042)	−0.014, 0.152	0.125 (0.118)	−0.106, 0.357	0.011 (0.122)	−0.229, 0.251
_X10_	Training (yes)	0.448 (0.161)**	0.132, 0.765	1.560 (0.450)**	0.677, 2.442	0.991 (0.466)*	0.078, 1.905
_X11_	Prior experience (yes)	0.106 (0.192)	−0.270, 0.483	−1.136 (0.536)*	−2.186, −0.086	0.039 (0.554)	−1.048, 1.125
_X12_	COVID-19 exposure (yes)	0.234 (0.151)	−0.062, 0.531	0.153 (0.422)	−0.674, 0.980	0.297 (0.437)	−0.559, 1.152
_X13_	Workplace stressors	0.025 (0.007)***	0.012, 0.038	0.050 (0.018)**	0.014, 0.085	0.056 (0.019)**	0.019, 0.092
_X14_	Cope	0.005 (0.024)	−0.042, 0.052	−0.069 (0.067)	−0.201, 0.063	−0.030 (0.070)	−0.167, 0.106
_X15_	Social support	−0.040 (0.030)	−0.100, 0.019	−0.226 (0.085)**	−0.392, −0.060	−0.217 (0.088)*	−0.388, −0.045
_X16_	Workplace support	−0.015 (0.008)∼	−0.030, 0.000	−0.071 (0.021)**	−0.112, −0.030	−0.080 (0.022)**	−0.123, −0.038

Note: ***p < .001; **p < .010; *p < .050, ∼*p* < .060.

### Mental Health Support

A large majority of respondents (*n* = 323, 85%) reported having accessed psychological support. Respondents most frequently sought psychological support from colleagues (74%) highlighting the importance of peer-support in healthcare settings. In addition, most reported that they had accessed psychological support during the past 2 years (85%, *n* = 323) with GPs and online information the most frequently accessed sources of help. Table 4 shows the types of support accessed including the frequency and satisfaction score for each. Accessing support via psychologists, colleagues and podcasts received the highest satisfaction rankings respectively.

**TABLE 4:** Psychological support accessed by healthcare staff (*n* = 431) Western Australia, 2021–2022.

Type of psychological support accessed^a^	N Accessed (%)	Satisfaction score (*M*, *SD*)
Colleague	278 (74%)	4.09 (0.95)
GP	157 (42%)	3.88 (1.13)
Online Information	146 (39%)	3.71 (1.38)
Podcast	123 (33%)	3.99 (0.75)
Psychologist	87 (23%)	4.21 (0.94)
Information Centres	80 (21%)	3.46 (0.93)
Mental Health App	60 (16%)	3.73 (1.01)
Employee Assistance Provider	53 (14%)	3.15 (0.94)
Staff Wellbeing Sessions	42 (11%)	3.29 (1.15)
Department of Health Hotline	28 (7.6%)	3.36 (1.16)
Mental Health Helplines	28 (7.6%)	3.29 (1.21)
Mental Health HUB	28 (7.6%)	3.25 (1.00)
Psychiatrist	25 (6.7%)	3.96 (1.06)
Check-in Sessions	22 (5.9%)	3.18 (1.40)
Telehealth Psych	18 (4.8%)	3.33 (1.14)
Professional Health Association	12 (3.2%)	3.67 (0.98)
Essential Network	7 (1.9%)	3.00 (0.58)
Other Services	38 (12%)	4.16 (0.95)

^a^ Does not add up to 100% as participants were allowed to click multiple options.

With regards to workplace supports, respondents reported an average satisfaction score of 35.1 (*SD* = 10.5, range 11–55, normally distributed) indicating moderately high levels of satisfaction when asked how satisfied they were with how the workplace supported their needs during the COVID-19 pandemic. The work support satisfaction score did not differ between professions, *F* (3,205) = 0.75, *p* = .525.

## Discussion

This study provides unique evidence for the mental health status of healthcare workers preparing for COVID-19 in a city that did not experience a major community outbreak until March 2022. Western Australian healthcare workers reported moderate levels of post-traumatic stress (22.3%), depressive (21.9%), and anxiety (25.9%) symptoms during the 3 months leading up to the re-opening of the state and international borders during the COVID-19 pandemic. Psychological symptom levels reported in Western Australia were consistent or slightly lower than assessments of healthcare workers in other Australian states [10, 35, 36], and substantially lower than pooled prevalence estimates reported in recent meta-analyses of global healthcare workers’ PTSS (31.4%–49%), depression (31.3%–40%) and anxiety (30%–37%) symptoms durings the COVID-19 pandemic [3, 37]. Smallwood et al.’ [10] assessment of psychological status among healthcare workers primarily located in Victoria, indicated higher levels of moderate to severe depression (28%) and moderate to severe anxiety (28.3%), likely reflecting the higher COVID-19 caseloads and more restrictive lockdowns in the eastern states of Australia. Western Australia’s successful implementation of border restrictions, rapid lockdowns and high vaccination rates before the first major COVID-19 outbreak may have supported resilience among healthcare staff. However, a sizeable minority of healthcare workers reported PTSS, depression and anxiety symptoms at moderate to severe levels, highlighting the impact of pre-existing and anticipatory stressors. Despite the lower levels of mental health difficulties in our sample, the mechanisms associated with psychological distress were consistent with the Australian and global literature [5, 6, 8–10, 38]. Workplace stressors such as increases to workload, a lack of timely communication, fear of infection, and concerns about access to personal protective equipment were significantly associated with higher levels of psychological symptoms, highlighting the importance of addressing structural workplace risks for mental health.

In partial support of Hypothesis One, mental health status differed by professional group. Doctors reported less severe PTSS than other disciplines, potentially reflecting a higher level of control over workplace factors, lower risk of moral injury, or reduced exposure to high-risk scenarios (including workplace violence and unexpected deaths) [39, 40]. Nursing roles were significantly associated with higher levels of PTSS. Higher levels of mental health concerns among nurses is consistent with research conducted prior to [41] and during [10] the COVID-19 pandemic from healthcare settings across Australia. The discrepancy may reveal the effects of longer periods spent attending to patients, higher risk of infection, and in some cases, threats of violence against nurses compared to other professions [40]. One third of the current sample reported exposure to physical violence at work, which highlights the need for safety improvements in the workplace for all staff, and nurses in particular [42]. The current findings indicated that executive and management staff reported high levels of PTSS, depression and anxiety, which has not been explored in prior studies of healthcare workers’ mental health, and warrants further investigation. Executive and management staff were under significant pressure to ensure that new COVID-19 policies and practices were implemented throughout the study period, while maintaining cost-efficiencies. Further, staff shortages due to border restrictions, rapid changes to staffing structures (including more junior personnel) and anticipatory stress among staff created additional concerns for management.

Workplace stressors including concerns about changes in policy, work hours, access to PPE, timely distribution of information, fear of infection, and fear of COVID-19 transmission to family members, were significantly associated with all mental health outcomes, supporting Hypothesis Two and augmenting findings on organizational stressors for healthcare workers in China [9], the United States [8], Italy [43], and Australia [38]. Despite the low COVID-19 caseload, the Western Australian health service implemented significant policy changes in preparation for the pandemic, creating additional workload and stressors for staff. Sleep difficulties were reported by half of the sample and were significantly associated with poorer mental health outcomes, lending support for Hypothesis Three. Healthcare workers employed in shift work or long hours are at greater risk of sleep-disorders [37], with implications for alertness, performance and mental health [44]. These factors are likely to be exacerbated during a pandemic [12, 37].

Hypothesis Four, that resilient coping, social support, infectious disease training and workplace supports would be associated with lower levels of PTSS, depressive and anxiety symptoms, was partially supported. The current findings demonstrated that social support and structural workplace supports (e.g., managerial support, timely and frequent information, access to sufficient PPE) were associated with lower depression and anxiety symptoms, signifying the importance of organizational support in the workplace to ensure psychological resilience among healthcare staff [38, 45, 46]. Resilient coping was not associated with mental health symptoms, and infectious disease training was positively associated with higher levels of PTSS, depressive and anxiety symptoms in contrast with prior research [16, 17]. It may be the case that staff with infectious disease training were more likely to be working in frontline roles, and were thus at greater risk of infection.

In addition, our findings indicated that younger healthcare workers were at higher risk of depression and anxiety, which may suggest the need for stronger mentoring processes and wellbeing supports tailored for early career professionals. Workforce instability in the healthcare system may disproportionately affect younger workers, creating higher levels of distress [39, 47]. Changes during the pandemic may have required younger staff to take on additional responsibilities, or step into roles previously held by senior staff who have left the workforce, highlighting a need for organizational support strategies to ensure emotional wellbeing among early career professionals. A large majority of our sample reported accessing mental health and wellbeing supports via colleagues, their general practitioner, or online, with high levels of satisfaction. Yet despite a growing focus on individual psychopathology among healthcare workers and high levels of access to psychosocial supports, the current findings contribute to a clear evidence base that argues the need for organizational change, including shorter shifts, collegial supports, access to effective PPE, and improved physical safety to support psychological health [11, 38, 46, 48].

### Policy and Practical Implications

It is critical that Health Emergency and Disaster Risk Management (Health EDRM) planning incorporates provisions for healthcare workers’ mental health and occupational support. To date, the World Health Organization’s Health EDRM Framework has focused on building capacity, core competencies, and skills within the existing healthcare workforce to address pandemics and disasters [49, 50]. The current findings suggest that attention to the mental health of healthcare staff is vital to ensure capacity to respond to large scale emergencies, and that organizational practices and mental health supports must be in place *prior* to the onset of mass trauma events. Pandemics are associated with higher levels of post-traumatic stress symptoms and anxiety than most other types of disasters [14]. Accordingly, greater investment in reducing exposure to infectious disease risk at work through the provision of PPE, ensuring timely communication of decisions, managerial responsiveness to feedback, and access to social and psychological supports in the workplace will enable health departments to retain staff throughout high-stress periods [11, 51]. Staff should also be confident in organisational disaster preparedness efforts [38]. Dedicated improvements to workplace culture are not only necessary during the COVID-19 pandemic, but vital to the ongoing functioning of the healthcare system.

### Limitations

This study presents the baseline data for a longitudinal study of mental health outcomes among tertiary hospital and community mental health clinic staff in Western Australia. The data were collected prior to Western Australia’s first major COVID-19 wave, but do not reflect ‘pre-pandemic’ mental health. Cross-sectional data provide an indication of potential risk and protective factors for mental health outcomes, but longitudinal data are needed to ascertain specific relationships over time. The sample comprised largely women (81%), reflecting gender imbalances in nursing and allied health [52]. Although the sample was inclusive of disciplines and roles within tertiary hospital settings and community mental health clinic settings, recruitment strategies and voluntary participation may have influenced representation within the sample, and thus the findings should not be generalised to all healthcare staff. Although we sought to recruit from four tertiary hospitals and a mental health clinic in metropolitan Perth, the rate of participation could not be determined due to the broad range of recruitment strategies used across different Health Service Providers, which limits the generalizability of findings. The proportion of doctors within the sample is low. Self-reported questionnaires do not provide a diagnostic assessment, and are likely to inflate the level of mental health need [53] and thus the current findings should be considered an indication of symptom levels among Western Australian healthcare workers. Further, we are unable to determine the extent to which adverse mental health outcomes reported in the current study reflect pandemic-related distress. Further follow-up is needed to elucidate novel COVID-induced stressors and the exacerbation of existing stressors.

### Conclusion

Despite Western Australia’s unique protection from COVID-19 for the first 2 years of the global pandemic, healthcare workers reported moderate symptom levels for PTSS, depression and anxiety. The current findings highlight the significant role of workplace stressors, younger age, nursing roles, and sleep difficulties in healthcare workers’ mental health. Importantly, most healthcare workers reported robust mental health, and social support and workplace supports played protective roles. Ongoing monitoring of healthcare workers’ mental health and wellbeing throughout the pandemic is needed, and initiatives to increase transparency in decision making, clear communication, and peer-based psychological support are vital. Organizational practices and mental health supports must be established prior to the onset of mass trauma events to ensure psychological resilience.
