# Supplementary material for: Mental Health Status, Risk and Protective Factors for Healthcare Staff Prior to the First Major COVID-19 Outbreak in Western Australia
Source: Int J Public Health. 2023 Sep 5;68:1606102. doi: 10.3389/ijph.2023.1606102 (PMC10507727; doi:10.3389/ijph.2023.1606102)

**Supplementary Material**

**Figure 1. Work Stressors Questionnaire**


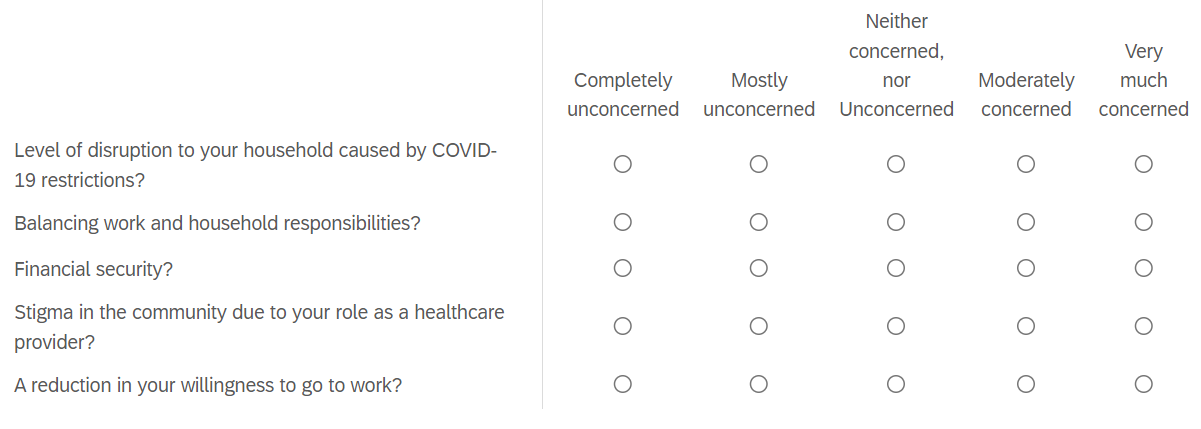

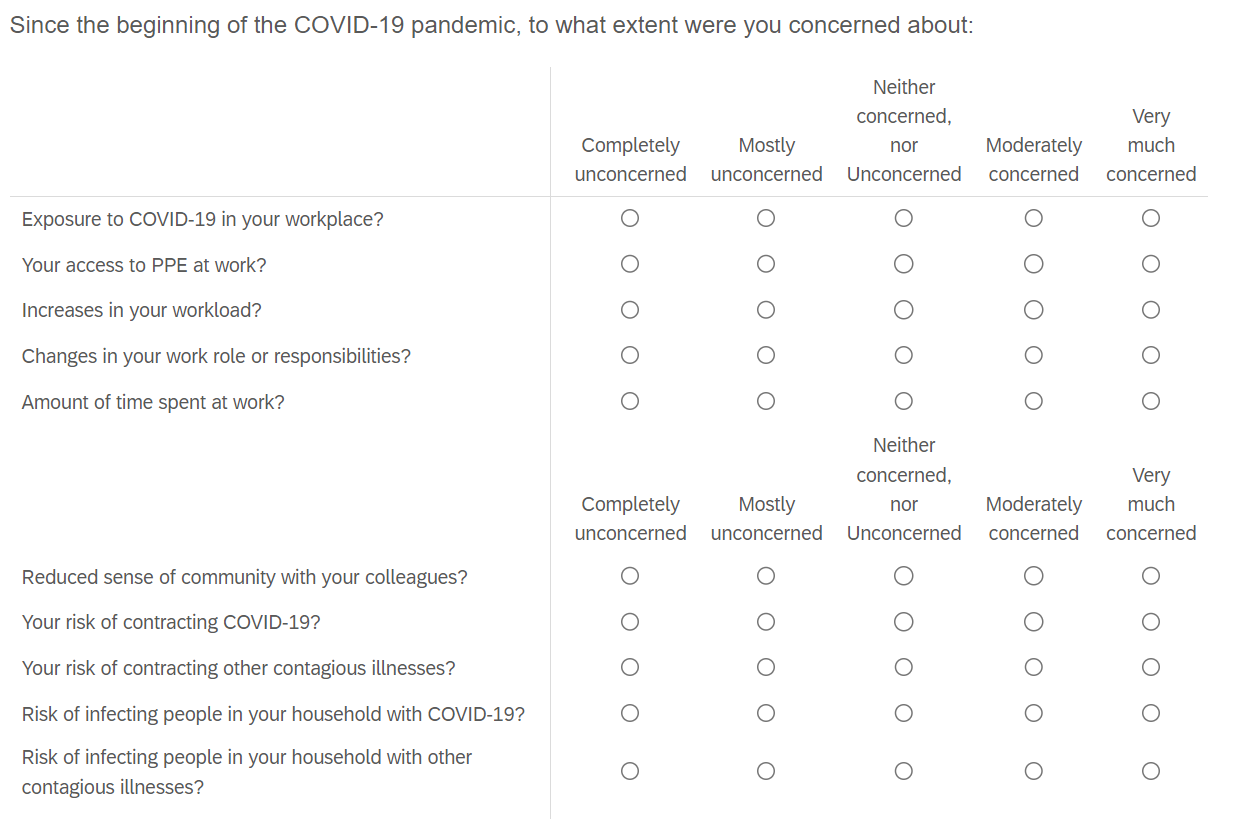


**Figure 2. Work Supports Questionnaire**


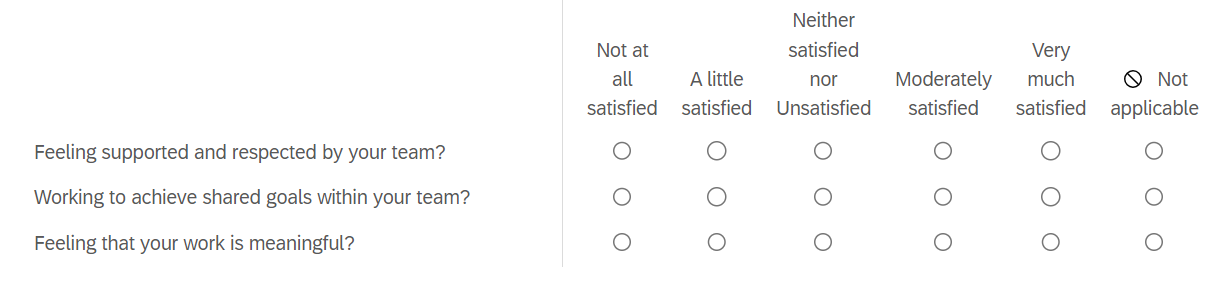

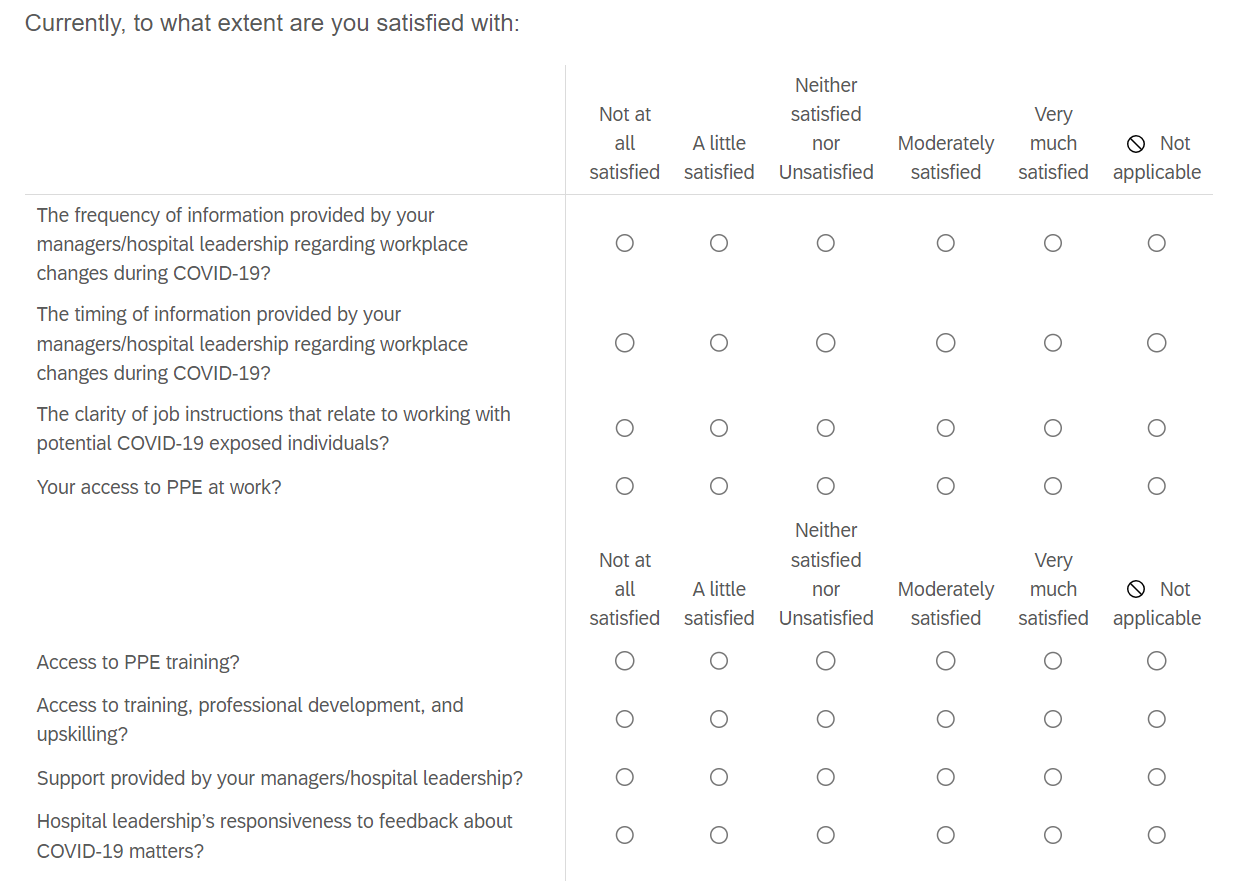


**Supplementary Table 1. Correlation matrix**

|  | PTSD | Depression | Anxiety | Cope | Social Support | Insomnia | Support Quality | Work Stress | Support Accessed | Age | Gender | Employment | Profession | Setting | Prior Experience | Training | Exposure | Work Support |
| --- | --- | --- | --- | --- | --- | --- | --- | --- | --- | --- | --- | --- | --- | --- | --- | --- | --- | --- |
| PTSD |  |  |  |  |  |  |  |  |  |  |  |  |  |  |  |  |  |  |
| Depression | .396^***^ |  |  |  |  |  |  |  |  |  |  |  |  |  |  |  |  |  |
| Anxiety | .458^***^ | .744^***^ |  |  |  |  |  |  |  |  |  |  |  |  |  |  |  |  |
| Cope | -.096 | -.196^***^ | -.170^***^ |  |  |  |  |  |  |  |  |  |  |  |  |  |  |  |
| Social Support | -.153^**^ | -.240^***^ | -.214^***^ | .267^***^ |  |  |  |  |  |  |  |  |  |  |  |  |  |  |
| Insomnia | .345^***^ | .528^***^ | .507^***^ | -.187^***^ | -.215^***^ |  |  |  |  |  |  |  |  |  |  |  |  |  |
| Support Quality | .266^***^ | .327^***^ | .293^***^ | .056 | -.066 | .275^***^ |  |  |  |  |  |  |  |  |  |  |  |  |
| Work Stress | .346^***^ | .349^***^ | .388^***^ | -.114^*^ | -.173^***^ | .396^***^ | .228^***^ |  |  |  |  |  |  |  |  |  |  |  |
| Support Accessed | .221^***^ | .190^***^ | .194^***^ | .015 | -.014 | .202^***^ | .505^***^ | .197^***^ |  |  |  |  |  |  |  |  |  |  |
| Age | -.075 | -.142^**^ | -.258^***^ | .070 | -.080 | -.066 | -.095 | -.168^**^ | -.007 |  |  |  |  |  |  |  |  |  |
| Gender | .047 | .142^**^ | .183^***^ | -.040 | .088 | .112^*^ | .142^**^ | .132^**^ | .169^**^ | -.173^***^ |  |  |  |  |  |  |  |  |
| Employment | .052 | .068 | .082 | -.001 | .020 | .098 | .113^*^ | -.005 | .054 | -.030 | -.069 |  |  |  |  |  |  |  |
| Profession | .142^**^ | .069 | .077 | -.051 | -.046 | .106^*^ | .028 | .214^***^ | .064 | -.103^*^ | .050 | -.160^***^ |  |  |  |  |  |  |
| Setting | .034 | .008 | .055 | -.024 | .034 | -.026 | -.098 | .026 | -.061 | -.131^**^ | .012 | -.069 | .230^***^ |  |  |  |  |  |
| Prior Experience | .105^*^ | -.012 | .028 | -.023 | -.049 | .083 | .083 | .076 | .038 | .109^*^ | -.086 | .106^*^ | .088 | -.030 |  |  |  |  |
| Training | .180^***^ | .163^***^ | .132^**^ | .020 | .037 | .127^*^ | .190^***^ | .108^*^ | .105^*^ | -.058 | .038 | .015 | .241^***^ | .061 | .240^***^ |  |  |  |
| COVID Exposure | .147^**^ | .041 | .097 | -.026 | -.021 | .041 | .048 | .066 | .046 | -.155^**^ | .019 | .107^*^ | .086 | .237^***^ | .158^**^ | .063 |  |  |
| Work Support | -.168^**^ | -.243^***^ | -.271^***^ | .117^*^ | .122^*^ | -.180^***^ | -.002 | -.245^***^ | -.013 | .022 | -.017 | -.038 | -.004 | .005 | .007 | .154^**^ | -.013 |  |
| Trauma Exposure | .182^**^ | .138^*^ | .088 | .060 | -.078 | .145^**^ | .107 | .120^*^ | .083 | -.034 | -.092 | .070 | .234^***^ | .162^**^ | .232^***^ | .210^***^ | .212^***^ | 019 |

*Note: *** p<.001; ** p<.010; *p<.050*

**Principal Components Analysis Output for the Workplace Stressors and Workplace Supports Questionnaires**


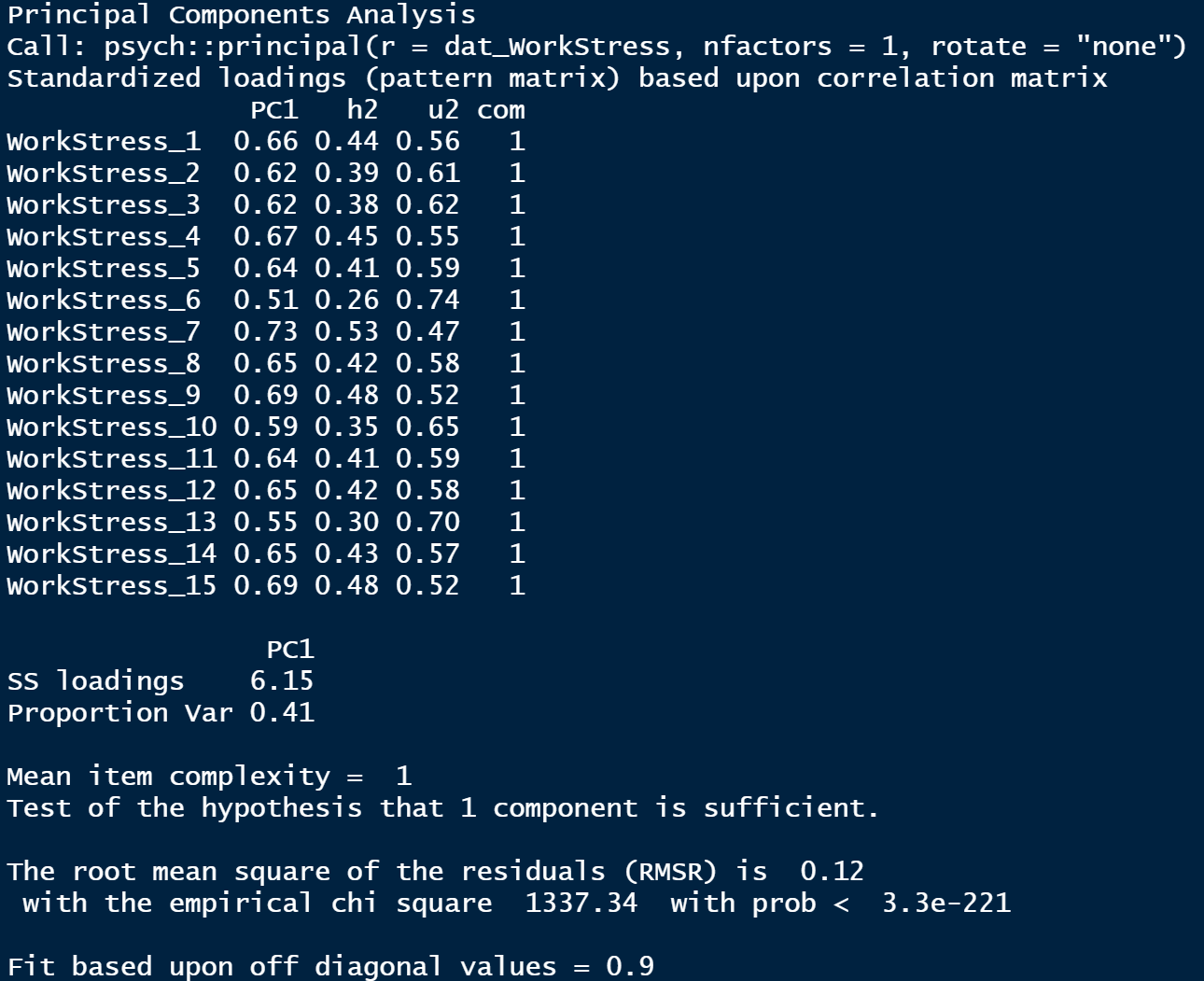


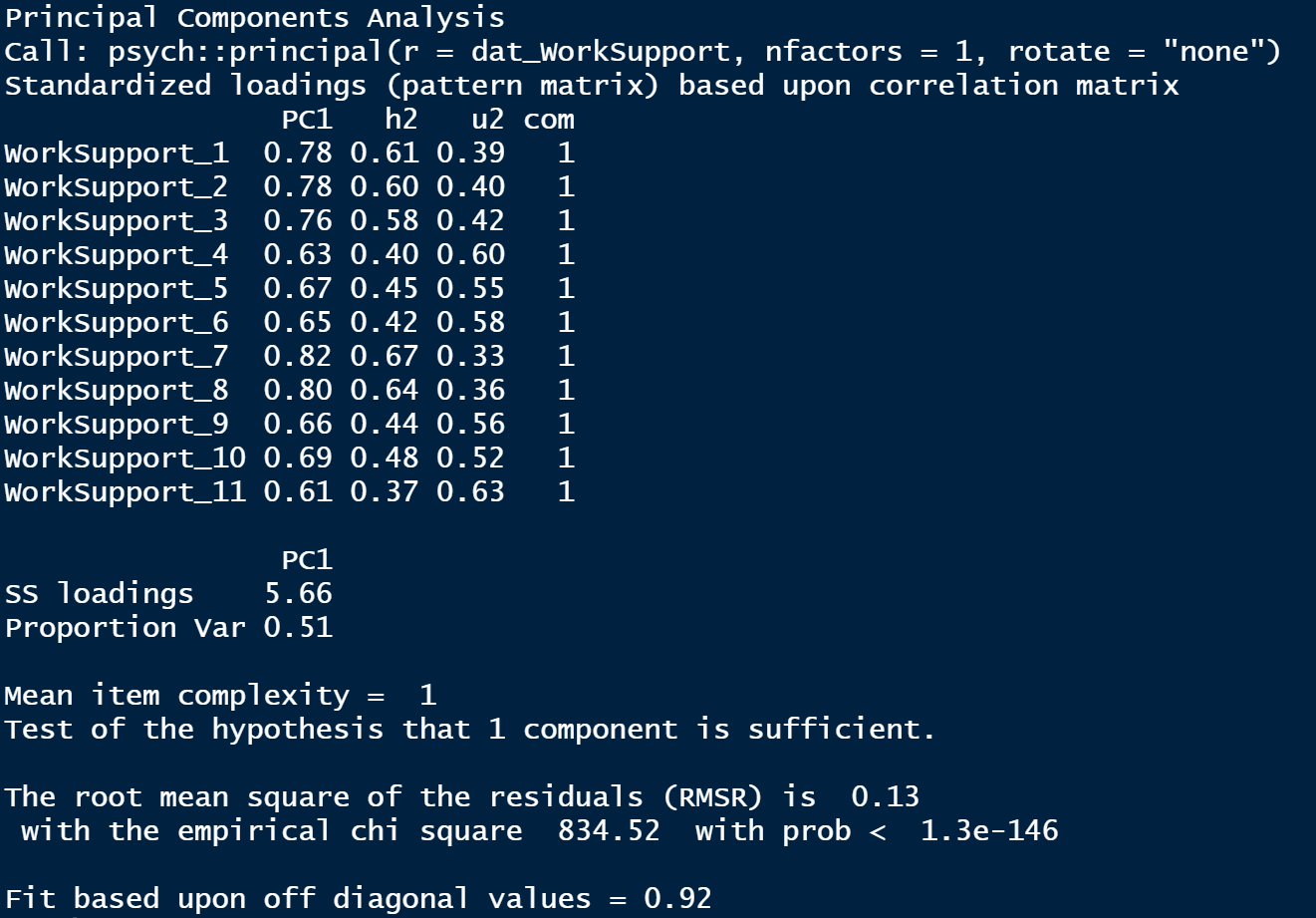

Supplement: Supplementary file 1 [file DataSheet1.docx]
